# Supplementary material for: The diagnosis and management of dehydration in children with wasting or nutritional edema: A systematic review
Source: PLOS Glob Public Health. 2023 Nov 3;3(11):e0002520. doi: 10.1371/journal.pgph.0002520 (PMC10624296; doi:10.1371/journal.pgph.0002520)
Supplement: S1 Checklist — (DOCX) [file pgph.0002520.s001.docx]

| **Section and Topic** | **Item #** | **Checklist item** | **Location where item is reported** |
| --- | --- | --- | --- |
| **TITLE** | | |  |
| Title | 1 | The Diagnosis and Management of Dehydration in Children with Wasting or Nutritional Edema: A Systematic Review | Page #1 |
| **ABSTRACT** | | |  |
| Abstract | 2 | Provide a structured summary including, as applicable: background; objectives; data sources; study eligibility criteria, participants, and interventions; study appraisal and synthesis methods; results; limitations; conclusions and implications of key findings.  **Background:** Dehydration is a major cause of death among children with wasting and diarrhea. We reviewed the evidence for the identification and management of dehydration among these children.  **Methods:** Two systematic reviews were conducted to assess 1) the diagnostic performance of clinical signs or algorithms intended to measure dehydration, and 2) the efficacy and safety of low-osmolarity ORS versus ReSoMal on mortality, treatment failure, time to full rehydration, and electrolyte disturbances (management review). We searched PubMed/Medline, Embase, and Global Index Medicus for studies enrolling children 0-60 months old with wasting and diarrhea.  **Findings:** The diagnostic review included four studies. Two studies found the Integrated Management of Childhood Illness (IMCI) and the Dehydration: Assessing Kids Accurately (DHAKA) algorithms had similar diagnostic performance, but both algorithms had high false positive rates for moderate (41% and 35%, respectively) and severe (76% and 82%, respectively) dehydration. One further IMCI algorithm study found a 23% false positive rate for moderate dehydration. The management review included six trials. One trial directly compared low osmolarity ORS to ReSoMal and found no difference in treatment failure rates, although ReSoMal had a shorter duration of treatment (16.1 vs. 19.6 hours, p=0.036) and a higher incidence of hyponatremia. Both fluids failed to correct a substantial number of hypokalemia cases across studies.  **Conclusion**: The IMCI dehydration assessment has comparable performance to other algorithms among wasted children. Low osmolarity ORS may be an alternative to ReSoMal for children with severe wasting, but might require additional potassium to combat hypokalemia. | Page #2 |
| **INTRODUCTION** | | |  |
| Rationale | 3 | Describe the rationale for the review in the context of existing knowledge, i.e., what is already known about your topic.  Dehydration is an important pathway leading to diarrhea-associated mortality^3^, and its identification and treatment remain the main focus of diarrhea management guidelines. The diagnosis of dehydration in children includes signs which are common among children with wasting, irrespective of their hydration status, indicating children with severe wasting may be frequently misdiagnosed with dehydration.  Low-osmolarity oral rehydration solution (ORS) is the cornerstone of diarrhea management, but guidelines recommend that children with severe wasting and dehydration (without shock or suspected cholera) be given Rehydration Solution for Malnourished (ReSoMal). It is unclear if ReSoMal is superior to low-osmolarity ORS for rehydrating children with severe wasting. | Page #3 |
| Objectives | 4 | Provide an explicit statement of the objective(s) or question(s) the review addresses with reference to participants, interventions, comparisons, outcomes, and study design (PICOS).  To summarize the evidence for the identification and management of dehydration among children with either moderate or severe wasting | Page #3&4 |
| **METHODS** | | |  |
| Eligibility criteria | 5 | Specify the inclusion and exclusion criteria for the review and how studies were grouped for the syntheses with study characteristics (e.g., PICOS, length of follow-up) and report characteristics (e.g., years considered, language, publication status) used as criteria for eligibility, giving rationale.  Inclusion criteria: abstracts, full-text articles, and pre-prints written in English, French, and Spanish; Studies that compare any clinical symptom/sign/test to named reference standard for dehydration; Studies comparing ReSoMal and WHO low-osmolarity ORS; No date restriction was applied – studied published until February, 2023 were included.  Exclusion criteria: studies focused on intravenous rehydration, or cholera/profuse watery diarrhea and those that did not use at least one of the solutions of interest (low-osmolarity ORS to ReSoMal | Page #4-6 |
| Information sources | 6 | Specify all databases, registers, websites, organisations, reference lists and other sources searched or consulted to identify studies. Specify the date when each source was last searched or consulted.  We searched PubMed/Medline, Embase, and Global Index Medicus | Page #3 |
| Search strategy | 7 | Present the full search strategies for all databases, registers and websites, including any filters and limits used.  We consulted a librarian to develop a search term for each data base and searched PubMed/Medline, Embase, and Global Index Medicus for abstracts, full-text articles, and pre-prints | Page# 3, Appendix #1 |
| Selection process | 8 | State the process for selecting studies (i.e., screening, eligibility).  Specify the methods used to decide whether a study met the inclusion criteria of the review, including how many reviewers screened each record and each report retrieved, whether they worked independently, and if applicable, details of automation tools used in the process.  Two authors (ATT, KDT) screened titles and abstracts for articles meeting the inclusion criteria. Disagreements were discussed, and when unresolved, a third author (PBP) held the decisive vote. | Page #5 |
| Study risk of bias assessment | 11 | Specify the methods used to assess risk of bias in the included studies, including details of the tool(s) used, how many reviewers assessed each study and whether they worked independently, and if applicable, details of automation tools used in the process.  Risk of bias was assessed using the QUADAS-2 tool for the diagnostic review. The management review used the Risk of Bias scale (RoB2). | Page #7 |
| **RESULTS** | | |  |
| Study selection | 16a | Describe the results of the search and selection process, from the number of records identified in the search to the number of studies included in the review, ideally using a flow diagram.  For the diagnosis review, of the 2,503 articles identified, 4 well included for full text review. For the management review, of the 1,712 articles, 6 were included for final full text review. | Page #8 & 12 |
|  | 16b | Cite studies that might appear to meet the inclusion criteria, but which were excluded, and explain why they were excluded. | Appendix 7 |
| Study characteristics | 17 | Cite each included study and present its characteristics (e.g., study size, PICOS, follow-up period). | Page #8-9 & #14-15 |
| Risk of bias in studies | 18 | Present assessments of risk of bias for each included study. | Appendix 3.1 & 3.2 |
| Results of individual studies | 19 | For all outcomes, present, for each study: (a) summary statistics for each group (where appropriate) and (b) an effect estimate and its precision (e.g. confidence/credible interval), ideally using structured tables or plots. |  |
| **DISCUSSION** | | |  |
| Discussion | 23a | Provide a general interpretation of the results in the context of other evidence. | Page #10 $ #14 |
|  | 23b | Discuss any limitations of the evidence included in the review. | Page #19 |
|  | 23c | Discuss any limitations of the review processes used. | Page #19 |
|  | 23d | Discuss implications of the results for practice, policy, and future research. | Page #19-20 |
| **OTHER INFORMATION** | | |  |
| Registration and protocol | 24a | Provide registration information for the review, including register name and registration number, or state that the review was not registered. | Page #3 |
|  | 24b | Indicate where the review protocol can be accessed, or state that a protocol was not prepared.  PROSPERO | Page #3 |
|  | 24c | Describe and explain any amendments to information provided at registration or in the protocol. | NA |
| Support | 25 | Describe sources of financial or non-financial support for the review, and the role of the funders or sponsors in the review. | Page #20 |
| Competing interests | 26 | Declare any competing interests of review authors. | Page #20 |
| Availability of data, code, and other materials | 27 | Report which of the following are publicly available and where they can be found: template data collection forms; data extracted from included studies; data used for all analyses; analytic code; any other materials used in the review. | All are publicly available and included in the study either as a supplementary document or a citation. |
