# Supplementary material for: The diagnosis and management of dehydration in children with wasting or nutritional edema: A systematic review
Source: PLOS Glob Public Health. 2023 Nov 3;3(11):e0002520. doi: 10.1371/journal.pgph.0002520 (PMC10624296; doi:10.1371/journal.pgph.0002520)
Supplement: S1 Text — (DOCX) [file pgph.0002520.s002.docx]

**Table A: Search terms for the diagnosis review**

**Embase; Elsevier**

|  | **Concept: Child** | **Concept: Malnutrition** | **Concept: Dehydration** | **Concept: Diagnosis** |
| --- | --- | --- | --- | --- |
| Subject Headings  (Emtree) | 'pediatrics'/exp  'child'/exp  'infant'/exp | 'nutritional disorder'/de  'malnutrition'/exp  'protein calorie malnutrition'/exp  'protein energy wasting'/exp  'nutritional status'/exp  'edema'/exp  'wasting syndrome'/exp | 'dehydration'/exp  'dehydration in infant and child'/exp  'dehydration in infancy and childhood'/exp  'hydration status'/exp | 'diagnosis'/exp  'diagnosis'/lnk |
| Free text terms  (searched in title, abstract, and keyword (:ti,ab,kw)) | infan*  newborn*  new-born*  baby  baby*  babies  toddler*  boy  boys  girl*  schoolchild*  child*:ti,ab,kw  p$ediatric*:ti,ab,kw  child*:jt  pediatric*:jt  paediatric*:jt | ((acute OR severe OR chronic OR protein) NEAR/2 malnutrition)  "nutritional status"  wasting  edema  oedema  kwas$io$kor  marasmus  "amino acid starvation"  "protein deprivation"  "protein starvation"  athrepsia | dehydrat*  "hydration status" | diagnos*  feature*  present*  detect* |

('pediatrics'/exp OR 'child'/exp OR 'infant'/exp OR infan* OR newborn* OR new-born* OR baby OR baby* OR babies OR toddler* OR boy OR boys OR girl* OR schoolchild* OR child*:ti,ab,kw OR p$ediatric*:ti,ab,kw OR child*:jt OR pediatric*:jt OR paediatric*:jt)

**AND**

('nutritional disorder'/de OR 'malnutrition'/exp OR 'protein calorie malnutrition'/exp OR 'protein energy wasting'/exp OR 'nutritional status'/exp OR 'edema'/exp OR 'wasting syndrome'/exp OR ((acute OR severe OR chronic OR protein) NEAR/2 malnutrition) OR "nutritional status" OR wasting OR edema OR oedema OR kwas$io$kor OR marasmus OR "amino acid starvation" OR "protein deprivation" OR "protein starvation" OR athrepsia)

**AND**

('dehydration'/exp OR 'dehydration in infant and child'/exp OR 'dehydration in infancy and childhood'/exp OR 'hydration status'/exp OR dehydrat* OR "hydration status")

**AND**

('diagnosis'/exp OR 'diagnosis'/lnk OR diagnos* OR feature* OR present* OR detect*)

**PubMed**

|  | **Concept: Child** | **Concept: Malnutrition** | **Concept: Dehydration** | **Concept: Diagnosis** |
| --- | --- | --- | --- | --- |
| Subject Headings  (MeSH) | "pediatrics"[Mesh]  "child"[Mesh]  "infant"[Mesh] | "Malnutrition"[Mesh]  "Child Nutrition Disorders"[Mesh]  "Protein-Energy Malnutrition"[Mesh]  "Nutritional Status"[Mesh]  "Edema"[Mesh]  "Wasting Syndrome"[Mesh] | "dehydration"[MeSH] | "Diagnosis"[Mesh]  "diagnosis" [Subheading] |
| Free text terms | infan*  newborn*  new-born*  baby  baby*  babies  toddler*  boy[tw]  boys[tw]  girl*  child*[tw]  child*[journal]  schoolchild*  pediatric*[tw]  pediatric*[journal]  paediatric*[tw]  paediatric*[journal] | “acute malnutrition”  “severe malnutrition”  “chronic malnutrition”  "nutritional status"  wasting  edema  oedema  kwashiorkor  kwashiokor  kwasiorkor  marasmus  “protein malnutrition”  “protein energy malnutrition”  “protein calorie malnutrition”  “amino acid starvation”  “protein deprivation”  “protein starvation”  athrepsia | Dehydrat*  “hydration status” | diagnos*  feature*  present*  detect* |

("pediatrics"[Mesh] OR "child"[Mesh] OR "infant"[Mesh] OR infan* OR newborn* OR new-born* OR baby OR baby* OR babies OR toddler* OR boy[tw] OR boys[tw] OR girl* OR child*[tw] OR child*[journal] OR schoolchild* OR pediatric*[tw] OR pediatric*[journal] OR paediatric*[tw] OR paediatric*[journal])

**AND**

("Malnutrition"[Mesh] OR "Child Nutrition Disorders"[Mesh] OR "Protein-Energy Malnutrition"[Mesh] OR "Nutritional Status"[Mesh] OR "Edema"[Mesh] OR "Wasting Syndrome"[Mesh] OR “acute malnutrition” OR “severe malnutrition” OR “chronic malnutrition” OR "nutritional status" OR wasting OR edema OR oedema OR kwashiorkor OR kwashiokor OR kwasiorkor OR marasmus OR “protein malnutrition” OR “protein energy malnutrition” OR “protein calorie malnutrition” OR “amino acid starvation” OR “protein deprivation” OR “protein starvation” OR athrepsia)

**AND**

("dehydration"[MeSH] OR Dehydrat* OR “hydration status”)

**AND**

("Diagnosis"[Mesh] OR "diagnosis" [Subheading] OR diagnos* OR feature* OR present* OR detect*)

**Global Index Medicus**

|  | **Concept: Child** | **Concept: Malnutrition** | **Concept: Dehydration** | **Concept: Diagnosis** |
| --- | --- | --- | --- | --- |
| Subject Descriptors | H02.403.670*  M01.060.406*  M01.060.703* | C18.654.521*  SP6.016.052.058*  C18.654.180*  C18.654.422*  C18.654.521.500.708.626*  SP6.016.052.058.089.005*  G07.203.650.650*  N01.224.425.525*  SP2.001.030.050*  SP2.036.010.024*  SP6.011*  C23.888.277*  C18.452.915*  C18.654.940* | C18.452.950.179*  C23.550.274* | E01*  SP4.051.512*  Q20* |
| Title, abstract, subject  (tw:()) | infan*  newborn*  new-born*  baby*  babies  toddler*  boy*  girl*  child*  schoolchild*  pediatric*  paediatric* | "acute malnutrition"  "severe malnutrition"  "chronic malnutrition"  "nutritional status"  wasting  edema  oedema  kwashiorkor  kwashiokor  kwasiorkor  marasmus  "protein malnutrition"  "protein energy malnutrition"  "protein calorie malnutrition"  "amino acid starvation"  "protein deprivation"  "protein starvation"  athrepsia | dehydrat*  "hydration status" | diagnos*  feature*  present*  detect* |

(H02.403.670* OR M01.060.406* OR M01.060.703* OR infan* OR newborn* OR new-born* OR baby* OR babies OR toddler* OR boy* OR girl* OR child* OR schoolchild* OR pediatric* OR paediatric*)

**AND**

(C18.654.521* OR SP6.016.052.058* OR C18.654.180* OR C18.654.422* OR C18.654.521.500.708.626* OR SP6.016.052.058.089.005* OR G07.203.650.650* OR N01.224.425.525* OR SP2.001.030.050* OR SP2.036.010.024* OR SP6.011* OR C23.888.277* OR C18.452.915* OR C18.654.940* OR "acute malnutrition" OR "severe malnutrition" OR "chronic malnutrition" OR "nutritional status" OR wasting OR edema OR oedema OR kwashiorkor OR kwashiokor OR kwasiorkor OR marasmus OR "protein malnutrition" OR "protein energy malnutrition" OR "protein calorie malnutrition" OR "amino acid starvation" OR "protein deprivation" OR "protein starvation" OR athrepsia)

**AND**

(C18.452.950.179* OR C23.550.274* OR dehydrat* OR "hydration status")

**AND**

(E01* OR SP4.051.512* OR Q20* OR diagnos* OR feature* OR present* OR detect*)

**Table B: Search terms for the management review**

**Embase; Elsevier**

|  | **Concept: child** | **Concept: Malnutrition** | **Concept: Dehydration/rehydration** | **Concept: Solution** |
| --- | --- | --- | --- | --- |
| Subject Headings  (Emtree) | 'pediatrics'/exp  'child'/exp  'infant'/exp | 'nutritional disorder'/de  'malnutrition'/exp  'protein calorie malnutrition'/exp  'protein energy wasting'/exp  'nutritional status'/exp  'edema'/exp  'wasting syndrome'/exp | 'dehydration'/exp  'dehydration in infant and child'/exp  'dehydration in infancy and childhood'/exp  'hydration status'/exp  'diarrhea'/exp  'rehydration'/exp | 'fluid therapy'/de  'rehydration'/exp  'oral rehydration solution'/exp  'rehydration solution'/exp  'world health organization oral rehydration solution'/exp |
| Free text terms  (searched in title, abstract, and keyword (:ti,ab,kw)) | infan*  newborn*  new-born*  baby  baby*  babies  toddler*  boy  boys  girl*  schoolchild*  child*:ti,ab,kw  p$ediatric*:ti,ab,kw  child*:jt  pediatric*:jt  paediatric*:jt | ((acute OR severe OR chronic OR protein) NEAR/2 malnutrition)  "nutritional status"  wasting  edema  oedema  kwas$io$kor  marasmus  "amino acid starvation"  "protein deprivation"  "protein starvation"  athrepsia | dehydrat*  "hydration status"  diarrh$ea  rehydrat* | "low-osmolarity ORS"  ReSoMal  "oral rehydration solution"  "WHO-ORS"  Gliukosolan  "hypo-osmolar oral rehydration solution"  "H-ORS" |

('pediatrics'/exp OR 'child'/exp OR 'infant'/exp OR infan* OR newborn* OR new-born* OR baby OR baby* OR babies OR toddler* OR boy OR boys OR girl* OR schoolchild* OR child*:ti,ab,kw OR p$ediatric*:ti,ab,kw OR child*:jt OR pediatric*:jt OR paediatric*:jt)

**AND**

('nutritional disorder'/de OR 'malnutrition'/exp OR 'protein calorie malnutrition'/exp OR 'protein energy wasting'/exp OR 'nutritional status'/exp OR 'edema'/exp OR 'wasting syndrome'/exp OR ((acute OR severe OR chronic OR protein) NEAR/2 malnutrition) OR "nutritional status" OR wasting OR edema OR oedema OR kwas$io$kor OR marasmus OR "amino acid starvation" OR "protein deprivation" OR "protein starvation" OR athrepsia)

**AND**

('dehydration'/exp OR 'dehydration in infant and child'/exp OR 'dehydration in infancy and childhood'/exp OR 'hydration status'/exp OR 'diarrhea'/exp OR 'rehydration'/exp OR dehydrat* OR "hydration status" OR diarrh$ea OR rehydrat*)

**AND**

('fluid therapy'/de OR 'rehydration'/exp OR 'oral rehydration solution'/exp OR 'rehydration solution'/exp OR 'world health organization oral rehydration solution'/exp OR "low-osmolarity ORS" OR ReSoMal OR "oral rehydration solution" OR "WHO-ORS" OR Gliukosolan OR "hypo-osmolar oral rehydration solution" OR "H-ORS")

1027 results as of 08/24/21

™

**PubMed**

|  | **Concept: child** | **Concept: Malnutrition** | **Concept: Dehydration/rehydration** | **Concept: Solution** |
| --- | --- | --- | --- | --- |
| Subject Headings  (MeSH) | "pediatrics"[Mesh]  "child"[Mesh]  "infant"[Mesh] | "Malnutrition"[Mesh]  "Child Nutrition Disorders"[Mesh]  "Infant Nutrition Disorders"[Mesh]  "Protein-Energy Malnutrition"[Mesh]  "Nutritional Status"[Mesh]  "Edema"[Mesh]  "Wasting Syndrome"[Mesh] | "dehydration"[MeSH] | **"Rehydration Solutions"[Mesh]**  **"Fluid Therapy"[Mesh:noexp]**  **"World Health Organization oral rehydration solution" [Supplementary Concept]** |
| Free text terms | infan*  newborn*  new-born*  baby  baby*  babies  toddler*  boy[tw]  boys[tw]  girl*  child*[tw]  child*[journal]  schoolchild*  pediatric*[tw]  pediatric*[journal]  paediatric*[tw]  paediatric*[journal] | “acute malnutrition”  “severe malnutrition”  “chronic malnutrition”  "nutritional status"  wasting  edema  oedema  kwashiorkor  kwashiokor  kwasiorkor  marasmus  “protein malnutrition”  “protein energy malnutrition”  “protein calorie malnutrition”  “amino acid starvation”  “protein deprivation”  “protein starvation”  athrepsia | dehydrat*  “hydration status”  diarrhea  rehydrat* | **"low-osmolarity ORS"**  **ReSoMal**  **"oral rehydration solution"**  **"WHO-ORS"**  **Gliukosolan**  **"hypo-osmolar oral rehydration solution"**  **"H-ORS"** |

**("pediatrics"[Mesh] OR "child"[Mesh] OR "infant"[Mesh] OR infan* OR newborn* OR new-born* OR baby OR baby* OR babies OR toddler* OR boy[tw] OR boys[tw] OR girl* OR child*[tw] OR child*[journal] OR schoolchild* OR pediatric*[tw] OR pediatric*[journal] OR paediatric*[tw] OR paediatric*[journal])**

**AND**

**("Malnutrition"[Mesh] OR "Child Nutrition Disorders"[Mesh] OR "Infant Nutrition Disorders"[Mesh] OR "Protein-Energy Malnutrition"[Mesh] OR "Nutritional Status"[Mesh] OR "Edema"[Mesh] OR "Wasting Syndrome"[Mesh] OR "acute malnutrition" OR "severe malnutrition" OR "chronic malnutrition" OR "nutritional status" OR wasting OR edema OR oedema OR kwashiorkor OR kwashiokor OR kwasiorkor OR marasmus OR "protein malnutrition" OR "protein energy malnutrition" OR "protein calorie malnutrition" OR "amino acid starvation" OR "protein deprivation" OR "protein starvation" OR athrepsia)**

**AND**

**("dehydration"[MeSH] OR dehydrat* OR "hydration status" OR diarrhea OR rehydrat*)**

**AND**

**("Rehydration Solutions"[Mesh] OR "Fluid Therapy"[Mesh:noexp] OR "World Health Organization oral rehydration solution" [Supplementary Concept] OR "low-osmolarity ORS" OR ReSoMal OR "oral rehydration solution" OR "WHO-ORS" OR Gliukosolan OR "hypo-osmolar oral rehydration solution" OR "H-ORS")**

319 results as of 8/24/21

**Global Index Medicus**

|  | **Concept: child** | **Concept: Malnutrition** | **Concept: Dehydration/rehydration** | **Concept: Solution** |
| --- | --- | --- | --- | --- |
| Subject Descriptors | H02.403.670*  M01.060.406*  M01.060.703* | C18.654.521*  SP6.016.052.058*  C18.654.180*  C18.654.422*  C18.654.521.500.708.626*  SP6.016.052.058.089.005*  G07.203.650.650*  N01.224.425.525*  SP2.001.030.050*  SP2.036.010.024*  SP6.011*  C23.888.277*  C18.452.915*  C18.654.940* | C18.452.950.179*  C23.550.274* | D26.776.741*  E02.319.360 |
| Title, abstract, subject  (tw:()) | infan*  newborn*  new-born*  baby*  babies  toddler*  boy*  girl*  child*  schoolchild*  pediatric*  paediatric* | "acute malnutrition"  "severe malnutrition"  "chronic malnutrition"  "nutritional status"  wasting  edema  oedema  kwashiorkor  kwashiokor  kwasiorkor  marasmus  "protein malnutrition"  "protein energy malnutrition"  "protein calorie malnutrition"  "amino acid starvation"  "protein deprivation"  "protein starvation"  athrepsia | dehydrat*  "hydration status"  diarrhea  rehydrat* | **"low-osmolarity ORS"**  **ReSoMal**  **"oral rehydration solution"**  **"WHO-ORS"**  **Gliukosolan**  **"hypo-osmolar oral rehydration solution"**  **"H-ORS"** |

(H02.403.670* OR M01.060.406* OR M01.060.703* OR infan* OR newborn* OR new-born* OR baby* OR babies OR toddler* OR boy* OR girl* OR child* OR schoolchild* OR pediatric* OR paediatric*)

**AND**

(C18.654.521* OR SP6.016.052.058* OR C18.654.180* OR C18.654.422* OR C18.654.521.500.708.626* OR SP6.016.052.058.089.005* OR G07.203.650.650* OR N01.224.425.525* OR SP2.001.030.050* OR SP2.036.010.024* OR SP6.011* OR C23.888.277* OR C18.452.915* OR C18.654.940* OR "acute malnutrition" OR "severe malnutrition" OR "chronic malnutrition" OR "nutritional status" OR wasting OR edema OR oedema OR kwashiorkor OR kwashiokor OR kwasiorkor OR marasmus OR "protein malnutrition" OR "protein energy malnutrition" OR "protein calorie malnutrition" OR "amino acid starvation" OR "protein deprivation" OR "protein starvation" OR athrepsia)

**AND**

(C18.452.950.179* OR C23.550.274* OR dehydrat* OR "hydration status" OR diarrhea OR rehydrat*)

**AND**

(D26.776.741* OR E02.319.360 OR "low-osmolarity ORS" OR ReSoMal OR "oral rehydration solution" OR "WHO-ORS" OR Gliukosolan OR "hypo-osmolar oral rehydration solution" OR "H-ORS")

Limits: 19 results as of 8/24/21

**Table C1: Risk of Bias Assessment using QUADAS 2.**

|  | **Risk of bias** | | | | **Concerns regarding applicability** | | |
| --- | --- | --- | --- | --- | --- | --- | --- |
|  | **Patient selection** | **Index test(s)** | **Reference standard** | **Flow and timing** | **Patient selection** | **Index test(s)** | **Reference standard** |
| **Beatty (1974)** | Low | Low | Unclear | Low | High | Low | High |
| **Nagpal (1992)** | High | Low | High | High | High | Low | High |
| **Skrable (2017)** | Low | Low | Unclear | High | Low | Low | Low |
| **Nijhawan (2020)** | Unclear | Low | High | High | Low | Low | High |

**Table C2: Risk of Bias assessment using the ROB2 tool for the available outcomes.** A full description of the ROBa assessment is given in Appendix 5.

| **Outcome** | **Study** | **Randomization** | **Deviations from interventions** | **Missing outcome data** | **Outcome measurement** | **Selection of reported results** | **Overall** |
| --- | --- | --- | --- | --- | --- | --- | --- |
| **Clinical detoriorations** | Kumar (2015) | Low | Low | Low | Some concerns^b^ | Low^c^ | Some concerns |
| **Time to full rehydration** | Kumar (2015) | Low | Some concerns^a^ | Some concerns^a^ | Some concerns^b^ | Low^c^ | High |
| **Mortality** | Kumar (2015) | Low | Some concerns^a^ | Some concerns^a^ | Low | Low^c^ | Some concerns |
| **Hyponatremia** | Kumar (2015) | Low | Some concerns^a^ | Low | Low | Low^c^ | Some concerns |
| **Hypernatremia** | Kumar (2015) | Low | Some concerns^a^ | Low | Low | Low^c^ | Some concerns |
| **Hypokalaemia** | Kumar (2015) | Low | Some concerns^a^ | Low | Low | Low^c^ | Some concerns |

^a^6 children excluded due to clinical deteriorations. These excluded children may have biased the time to full rehydration outcome, as the duration of their treatment is not reported in the paper. This bias is unlikely to effect other results in the trial.

^b^Trial was open label, suggesting outcomes without objective measurements could have been influenced by knowledge of the treatment.

^c^The clinical trial record for this study did say they would report on vomiting incidence across the arms, which was not discussed in their manuscript. We did not penalize the manuscript for this omission as all other outcomes were reported.

The critical weakness across all areas were that the only direct comparison between the two rehydration formulations was a small, open label study. While the study that compared the two solutions directly (Kumar 2015) was well designed, the ROB2 tool identified two possible biases that may have influenced the results. Firstly, six children who developed clinical deteriorations were excluded from all results except those pertaining to treatment failure/success, and this may have influenced the outcomes from which these children were excluded, particularly time to full rehydration. Additionally, this was an open label trial, which may suggest that the assessment of more subjective outcomes, such as time to full rehydration, could be vulnerable to bias.

**Table C3: ROB2 assessment of the included trial**

|  | **Clinical deteriorations** | **Time to full rehydration** | **Mortality** | **Hyponatremia** | **Hypernatremia** | **Hypokalaemia** |
| --- | --- | --- | --- | --- | --- | --- |
| **Randomization** |  |  |  |  |  |  |
| 1.1 Was the allocation sequence random? | Y | Y | Y | Y | Y | Y |
| 1.2 Was the allocation sequence concealed until participants were enrolled and assigned to interventions? | PY | PY | PY | PY | PY | PY |
| 1.3 Did baseline differences between intervention groups suggest a problem with the randomization process? | N | N | N | N | N | N |
| **Risk of Bias Judgement** | **Low** | **Low** | **Low** | **Low** | **Low** | **Low** |
| **Deviations from interventions** |  |  |  |  |  |  |
| 2.1. Were participants aware of their assigned intervention during the trial? | Y: open label | Y: open label | Y: open label | Y: open label | Y: open label | Y: open label |
| 2.2. Were carers and people delivering the interventions aware of participants' assigned intervention during the trial? | Y: open label | Y: open label | Y: open label | Y: open label | Y: open label | Y: open label |
| 2.3. If Y/PY/NI to 2.1 or 2.2: Were there deviations from the intended intervention that arose because of the trial context? | PN | Y: 3/arm excluded | Y: 3/arm excluded | Y: 3/arm excluded | Y: 3/arm excluded | Y: 3/arm excluded |
| 2.4 If Y/PY to 2.3: Were these deviations likely to have affected the outcome? | N: deviations were the outcome | Y | Y | Y | Y | Y |
| 2.5. If Y/PY/NI to 2.4: Were these deviations from intended intervention balanced between groups? | Y | Y | Y | Y | Y | Y |
| 2.6 Was an appropriate analysis used to estimate the effect of assignment to intervention? | Y | PY | PY | PY | PY | PY |
| 2.7 If N/PN/NI to 2.6: Was there potential for a substantial impact (on the result) of the failure to analyse participants in the group to which they were randomized? | -- | -- | -- | -- | -- | -- |
| **Risk-of-bias judgement** | **Low** | **Some concerns** | **Some concerns** | **Some concerns** | **Some concerns** | **Some concerns** |
| **Missing outcome data** |  |  |  |  |  |  |
| 3.1 Were data for this outcome available for all, or nearly all, participants randomized? | Y | N: 3/arm excluded | N: 3/arm excluded | N: 3/arm excluded | N: 3/arm excluded | N: 3/arm excluded |
| 3.2 If N/PN/NI to 3.1: Is there evidence that the result was not biased by missing outcome data? | -- | PN | PN | PN | PN | PN |
| 3.3 If N/PN to 3.2: Could missingness in the outcome depend on its true value? | -- | PY: Shocked children longer rehydration | PY: Shocked children higher mortality | PN | PN | PN |
| 3.4 If Y/PY/NI to 3.3: Is it likely that missingness in the outcome depended on its true value? | -- | PN | PN | -- | -- | -- |
| **Risk-of-bias judgement** | **Low** | **Some concerns** | **Some concerns** | **Low** | **Low** | **Low** |
| **Outcome measurement** |  |  |  |  |  |  |
| 4.1 Was the method of measuring the outcome inappropriate? | N | PN | N | N | N | N |
| 4.2 Could measurement or ascertainment of the outcome have differed between intervention groups? | Y: subjective measure | Y: subjective measure | N: objective measure | N: objective measure | N: objective measure | N: objective measure |
| 4.3 If N/PN/NI to 4.1 and 4.2: Were outcome assessors aware of the intervention received by study participants? | Y: open label | Y: open label | Y: open label | Y: open label | Y: open label | Y: open label |
| 4.4 If Y/PY/NI to 4.3: Could assessment of the outcome have been influenced by knowledge of intervention received? | Y: subjective | Y: subjective | N: objective | N: objective | N: objective | N: objective |
| 4.5 If Y/PY/NI to 4.4: Is it likely that assessment of the outcome was influenced by knowledge of intervention received? | PN | PN | N | N | N | N |
| **Risk-of-bias judgement** | **Some concerns** | **Some concerns** | **Low** | **Low** | **Low** | **Low** |
| **Selection of reported results** |  |  |  |  |  |  |
| 5.1 Were the data that produced this result analysed in accordance with a pre-specified analysis plan that was finalized before unblinded outcome data were available for analysis? | PY – retrospective registration | PY – retrospective registration | PY – retrospective registration | PY – retrospective registration | PY – retrospective registration | PY – retrospective registration |
| Is the numerical result being assessed likely to have been selected, on the basis of the results, from... |  |  |  |  |  |  |
| 5.2. ... multiple eligible outcome measurements (e.g. scales, definitions, time points) within the outcome domain? | PN: vomiting result omitted, otherwise complete reporting | PN: vomiting result omitted, otherwise complete reporting | PN: vomiting result omitted, otherwise complete reporting | PN: vomiting result omitted, otherwise complete reporting | PN: vomiting result omitted, otherwise complete reporting | PN: vomiting result omitted, otherwise complete reporting |
| 5.3 ... multiple eligible analyses of the data? | PN | PN | PN | PN | PN | PN |
| **Risk-of-bias judgement** | **Low** | **Low** | **Low** | **Low** | **Low** | **Low** |

**Table D: Dehydration diagnosis composite scores**

| **Clinical manifestations** | **WHO IMCI**  **(for all children)** | | **CDS** | **DHAKA** | **Beatty (1974)** |
| --- | --- | --- | --- | --- | --- |
|  | **Category 1** | **Category 2** |  |  |  |
| **General appearance** | lethargic/unconscious | Restless/irritable | 0 = Normal  1= Thirsty, restless, irritable  2= Lethargic unconscious | 0 = Normal  1= Thirsty, restless, irritable  2= Lethargic unconscious |  |
| **Eyes** | Sunken eyes | Sunken eyes | 0= Normal  1= Slightly Sunken  2= Very sunken |  | Sunken eyes |
| **Tears** |  |  | 0= Tears present  1= Decreased tears  2= Absent tears | 0= Tears present  2= Decreased tears  4= Absent tears |  |
| **Respiration** |  |  |  | 0= Normal  2= Deep |  |
| **Drinking** | Not able to drink or drink poorly | Thirsty/drink eagerly |  |  |  |
| **Mucus membrane** |  |  | 0= Moist  1= Dry  2= Very dry |  | Dry mucous membranes |
| **Fontanelle** |  |  |  |  | Sunken fontanelle |
| **Skin pinch** | Very slow | Slow |  | 0= Normal  2= Slow  4= Very slow | Loss of tissue turgor |
| ***Severe dehydration*** | ***2 of the signs*** |  | ***5-8*** | ***>=4*** | ***1 of the signs*** |
| ***Some dehydration*** |  | ***2 of the signs*** | ***1-4*** | ***2-3*** | ***2 of the signs*** |
| ***No dehydration*** | ***No enough signs to classify either some or severe*** | | ***0*** | ***0-1*** |  |
| ***Studies that used the method*** | - Scrable (2017) - Nijhawan (2020) - Nagpal (1992) | | - Scrable (2017) | - Scrable (2017) - Nijhawan (2020) | - Beatty (1974) |

**Table E. Grade Evidence profile for the diagnostic review**

**Table E1: Comparison 1: GRADE Evidence Profile for diagnostic test assessment for the WHO IMCI algorithm, DHAKA and CDS scores compared to change in pre and post rehydration weight for identifying ANY dehydration among wasted children**

| **Question** | **What is the diagnostic accuracy of WHO IMCI, DHAKA and CDS scores for the diagnosis of ANY dehydration (weight change >=3%)?** | | | | | | | | |
| --- | --- | --- | --- | --- | --- | --- | --- | --- | --- |
| **Population** | Children aged 0-59 months, with moderate or severe wasting or edema or growth faltering and dehydration | | | | | | | | |
| **Index tests** | WHO IMCI algorithm (only Some dehydration = 2 signs in Cat 2)  DHAKA score **(**≥2 = Some and Severe dehydration)  CDS scale (from No [0] to Some [1-4] to Severe [5-8]) | | | | | | | | |
| **Target condition** | Dehydration associated with diarrhea | | | | | | | | |
| **Reference standard** | Change in pre and post rehydration weight (**any** dehydration, >=3% weight change) | | | | | | | | |
| **Action** | **Low sensitivity:** failure to detect and treat dehydration  Consequences: electrolyte imbalance, shock, end organ damage, longer hospital admission, and death  Clinically important | | | | | | **Low specificity:** incorrectly classification of children as dehydrated and unnecessary treatment with ORS/ReSoMal  Consequences: electrolyte imbalance, fluid overload, heart failure/pulmonary edema, and death  Possibly less clinically important than low sensitivity (lower risk of consequences, as it is more dangerous to miss a child with dehydration) | | |
| **Summary** | \| **Test/score** \| **Sensitivity (95% CI)** \| **Specificity (95% CI)** \| **Area under the curve across (not specific to moderate or severe dehydration)** \| **Prevalence** \| \| --- \| --- \| --- \| --- \| --- \| \| WHO IMCI algorithm (only Some dehydration = 2 signs in Cat 2) \| 0.97 (0.94-1.00), \| 0.15 (0.08-0.22) \| 0.713 (0.659-0.768) \| 56% \| \| DHAKA **(**≥2 = Some and Severe dehydration) \| 0.88 (0.83-0.94) \| 0.40 (0.30-0.49) \| 0.783 (0.723-0.843) \| \| CDS (from No [0] to Some [1-4] to Severe [5-8]) \| Not reported \| Not reported \| 0.774 (0.714-0.834) \| | | | | | | | | |
| **Outcome** | **№ of studies (№ of patients)** | **Study design** | **Factors that may decrease certainty of evidence** | | | | | **Effect per 1,000 patients tested (56% prevalence)** | **Test accuracy Certainty of Evidence** |
|  |  |  | **Risk of bias^a^** | **Indirectness^b^** | **Inconsistency** | **Imprecision** | **Publication bias** |  |  |
| **IMCI algorithm Some dehydration (2 signs in Cat 2) versus Change in pre and post rehydration weight (any dehydration, >=3% weight change)** | | | | | | | | | |
| **Sensitivity: 0.97 (0.94-1.00), Specificity: 0.15 (0.08-0.22). Prevalence: 56%** | | | | | | | | | |
| **True positives** (patients with dehydration) | 1 study 134 patients  (Skrable 2017) | Cross-sectional (cohort type) | Serious | Serious | Not serious | Serious^c^ | Untested | 543 (526 to 560) | Very Low ^a, b, c^ |
| **False negatives** (patients incorrectly classified as not having dehydration) |  |  |  |  |  |  |  | 17 (0 to 34) |  |
| **True negatives** (patients without dehydration) | 1 study 106 patients  (Skrable 2017) | Cross-sectional (cohort type) | Serious | Serious | Not serious | Serious^c^ | Untested | 66 (35 to 97) | Very Low ^a, b, c^ |
| **False positives** (patients incorrectly classified as having dehydration) |  |  |  |  |  |  |  | 374 (343 to 405) |  |
| **DHAKA scale ≥2 Some and Severe dehydration versus Change in pre and post rehydration weight (any dehydration, >=3% weight change)** | | | | | | | | | |
| **Sensitivity: 0.88 (0.83-0.94), Specificity: 0.40 (0.30-0.49). Prevalence: 56%** | | | | | | | | | |
| **True positives** (patients with dehydration) | 1 study 134 patients  (Skrable 2017) | Cross-sectional (cohort type) | Serious | Serious | Not serious | Serious^c^ | Untested | 493 (465 to 526) | Very Low ^a, b, c^ |
| **False negatives** (patients incorrectly classified as not having dehydration) |  |  |  |  |  |  |  | 67 (34 to 95) |  |
| **True negatives** (patients without dehydration) | 1 study 106 patients  (Skrable 2017) | Cross-sectional (cohort type) | Serious | Serious | Not serious | Serious^c^ | Untested | 176 (132 to 216) | Very Low ^a, b, c^ |
| **False positives** (patients incorrectly classified as having dehydration) |  |  |  |  |  |  |  | 264 (224 to 308) |  |
| **CDS (from 0 to 8) versus Change in pre and post rehydration weight (any dehydration, >=3% weight change)** | | | | | | | | | |
|  | 1 study  (Skrable 2017) | Cross-sectional (cohort type) | Serious | Serious | Not serious | Serious^d^ | Not serious | AUC:0.774 (0.714-0.834) | Very Low ^a, b, d^ |

AUC: area under the curve

1. Serious risk of bias: High risk of bias for flow and timing domain for reference standard domain. The index tests were done upon admission, and the reference standard was done after the patients were treated. There was a time gap between the two measures- from admission until patients developed a stable weight. But the gap was different across patients depending on how fast they had a stable weight. Some achieved it before hospital discharge and others after discharge. In between, out of the 1396 participants, 113 (8%) were excluded due to lacking a final stable weight data.
2. Serious indirectness: There was only one study in this comparison, conducted in Bangladesh. Applicability to other settings comes with some uncertainty. The reference standard was a 3% change in pre and post rehydration weight, which does not align with the 5% typically used by WHO.
3. Serious imprecision: The number of participants with wasting was very small – 58 severely wasted and 182 moderately wasted.
4. Serious imprecision: The number of participants with wasting was very small – 58 severely wasted and 182 moderately wasted. Sensitivity and specificity are not reported.

**Table E2: Comparison 2: GRADE Evidence Profile for diagnostic test assessment for the WHO IMCI algorithm, DHAKA and CDS scores compared to change in pre and post rehydration weight for identifying SEVERE dehydration among wasted children**

| **Question** | **What is the diagnostic accuracy of WHO IMCI, CDS, and DHAKA scores for the diagnosis of SEVERE dehydration (weight change > 9%)?** | | | | | | | | | | | | | |
| --- | --- | --- | --- | --- | --- | --- | --- | --- | --- | --- | --- | --- | --- | --- |
| **Population** | Children aged 0-59 months, with moderate or severe wasting or edema or growth faltering and dehydration | | | | | | | | | | | | | |
| **Index tests** | WHO IMCI algorithm (only Severe dehydration = 2 signs in Cat 1)  DHAKA score ≥4 (only Severe)  CDS scale (from no [0] to Some [1-4] to Severe [5-8]) | | | | | | | | | | | | | |
| **Target condition** | Dehydration associated with diarrhea | | | | | | | | | | | | | |
| **Reference standard** | Change in pre and post rehydration weight (severe dehydration, > 9% weight change) | | | | | | | | | | | | | |
| **Action** | **Low sensitivity:** failure to detect and treat dehydration.  Consequences: electrolyte imbalance, shock, end organ damage, longer hospital admission, and death  Clinically important | | | | | | | | | **Low specificity:** incorrectly classification of children as dehydrated and unnecessary treatment with ORS/ReSoMal  Consequences: electrolyte imbalance, fluid overload, heart failure/pulmonary edema, and death  Clinical importance unclear.^a^ | | | | |
| **Summary** | \| **Test/score** \| **Sensitivity (95% CI)** \| **Specificity (95% CI)** \| **Area under the curve (not specific to moderate or severe dehydration)** \| **Prevalence** \| \| --- \| --- \| --- \| --- \| --- \| \| WHO IMCI algorithm (only Severe dehydration = 2 signs in Cat 1) \| 0.76 (0.83-0.94) \| 0.72 (0.66-0.78) \| 0.713 (0.659-0.768) \| 10% \| \| Dhaka ≥4 (only Severe) \| 0.88 (0.75-1.00) \| 0.55 (0.48-0.62) \| 0.783 (0.723-0.843) \| \| CDS (from no [0] to Some [1-4] to Severe [5-8]) \| Not reported \| Not reported \| AUC:0.774 (0.714-0.834) \| | | | | | | | | | | | | | |
|  | **№ of studies (№ of patients)** | | **Study design** | **Factors that may decrease certainty of evidence** | | | | | | | **Effect per 1,000 patients tested (10% prevalence)** | | | **Test accuracy CoE** |
|  |  |  |  | **Risk of bias^b^** | | **Indirectness^c^** | **Inconsistency** | | **Imprecision** | **Publication bias** |  |  |  |  |
| **IMCI algorithm severe dehydration (only Severe dehydration = 2 signs in Cat 1) versus Change in pre and post rehydration weight severe dehydration (>9% weight change)** | | | | | | | | | | | | | | |
| **Sensitivity: Specificity: Prevalence: 10%** | | | | | | | | | | | | | | |
| **True positives** (patients with dehydration) | | 1 study 25 patients  (Skrable 2017) | Cross-sectional (cohort type) | Serious | Serious | | -- | Serious^d^ | | Untested | | 76 (59 to 93) | Very Low ^a, b, c^ | |
| **False negatives** (patients incorrectly classified as not having dehydration) | |  |  |  |  |  |  |  |  |  |  | 24 (7 to 41) |  |  |
| **True negatives** (patients without dehydration) | | 1 study 215 patients  (Skrable 2017) | Cross-sectional (cohort type) | Serious | Serious | | -- | Serious^d^ | | Untested | | 648 (594 to 702) | Very Low ^a, b, c^ | |
| **False positives** (patients incorrectly classified as having dehydration) | |  |  |  |  |  |  |  |  |  |  | 252 (198 to 306) |  |  |
| **False positives** (patients incorrectly classified as having dehydration) | |  |  |  |  |  |  |  |  |  |  | 264 (224 to 308) |  |  |
| **DHAKA scale (≥4, severe dehydration) versus Change in pre and post rehydration weight severe dehydration (> 9% weight change)** | | | | | | | | | | | | | | |
| **Sensitivity: 0.88 (0.75-1.00), Specificity: 0.55 (0.48-0.62). Prevalence: 10%** | | | | | | | | | | | | | | |
| **True positives** (patients with dehydration) | | 1 study 25 patients  (Skrable 2017) | Cross-sectional (cohort type) | Serious | Not serious | | Not serious | Serious^d^ | | Not serious | | 88 (75 to 100) | Very Low  ^b, c, d^ | |
| **False negatives** (patients incorrectly classified as not having dehydration) | |  |  |  |  |  |  |  |  |  |  | 12 (0 to 25) |  |  |
| **True negatives** (patients without dehydration) | | 1 study 215 patients  (Skrable 2017) | Cross-sectional (cohort type) | Serious | Serious | | Not serious | Serious^d^ | | Not serious | | 495 (432 to 558) | Very Low  ^b, c, d^ | |
| **False positives** (patients incorrectly classified as having dehydration) | |  |  |  |  |  |  |  |  |  |  | 405 (342 to 468) |  |  |
| **CDS (from 0 to 8) versus Change in pre and post rehydration weight (severe dehydration, > 9% weight change)** | | | | | | | | | | | | | | |
|  | | 1 study  (Skrable 2017) , | Cross-sectional (cohort type) | Serious | Serious | | Not serious | Serious^d^ | | Not serious | | AUC:0.774 (0.714-0.834) | Very Low  ^b, c, d^ | |

AUC: Area under the curve

1. Recent evidence has suggested that wasted children may be less vulnerable to fluid overload than previous thought, although they are still at risk of electrolyte imbalances.
2. Serious risk of bias: High risk of bias for flow and timing domain for reference standard domain. The index tests were done upon admission, and the reference standard was done after the patients were treated. There was a time gap between the two measures- from admission until patients developed a stable weight. But the gap was different across patients depending on how fast they had a stable weight. Some achieved it before hospital discharge and others after discharge. In between, out of the 1396 participants, 113 (8%) were excluded due to lacking a final stable weight data.
3. Serious indirectness: There was only one study in this comparison, conducted in Bangladesh. Applicability to other settings comes with some uncertainty. The reference standard was a 3% change in pre and post rehydration weight, which does not align with the 5% typically used by WHO.
4. Serious imprecision: The number of participants with wasting was very small – 58 severely wasted and 182 moderately wasted. Sensitivity and specificity are not reported.

**Table E3: Comparison 3: GRADE Evidence Profile for diagnostic test assessment for the DHAKA score compared to WHO IMCI algorithm for identifying dehydration among wasted children**

| **Question** | **What is the diagnostic accuracy of DHAKA compared to IMCI scores for the diagnosis of dehydration?** | | | | | | | | |  |
| --- | --- | --- | --- | --- | --- | --- | --- | --- | --- | --- |
| **Population** | Children aged 0-59 months, with moderate or severe wasting or edema or growth faltering and dehydration | | | | | | | | |  |
| **Index tests** | DHAKA score (from No [0-1] to Some [2-3] to Severe [>=4]) | | | | | | | | |  |
| **Target condition** | Dehydration associated with diarrhea | | | | | | | | |  |
| **Reference standard** | WHO IMCI algorithm | | | | | | | | |  |
| **№ of studies (№ of patients)** | | **Study design** | **Factors that may decrease certainty of evidence** | | | | | **Effect Estimate** | **Confidence** | |
|  |  |  | **Risk of bias** | **Indirectness** | **Inconsistency** | **Imprecision** | **Publication bias** |  |  |  |
|  | | | | | | | | | | |
| AUC of DHAKA score (from 0 to >=4) against WHO IMCI algorithm for DEHYDRATION | | | | | | | | | | |
| 1 study  (Nijhawan 2020) | | Cross-sectional (cohort type)  N= 503 | Serious^a^ | Not serious | Not serious | Not serious | Untested^b^ | AUC: 0.92  Sensitivity: 84%  Specificity: 100%  (95% CIs no reported) | Very Low | |

a. Only the abstract is available, therefore the method of patient selection, who received the index test or the reference standard or both, and the time interval and the presence of any intervention between the index test and the reference standard are unclear or unknown.

b. No tests for small study or publication bias were conducted since it was the only study identified. GRADE guidance suggests we do not assume publication bias if only one study exists and we believe the search we conducted have been thorough.

**Table E4: Comparison 4: GRADE Evidence Profile for diagnostic test assessment for the WHO IMCI algorithm compared to pre- and post-rehydration weight change for identifying MODERATE dehydration among wasted children**

| **Question** | **What is the diagnostic accuracy of DHAKA compared to IMCI scores for the diagnosis of dehydration?** | | | | | | | | |  |
| --- | --- | --- | --- | --- | --- | --- | --- | --- | --- | --- |
| **Population** | Children aged 0-59 months, with moderate or severe wasting or edema or growth faltering and dehydration | | | | | | | | |  |
| **Index tests** | WHO IMCI algorithm | | | | | | | | |  |
| **Target condition** | Dehydration associated with diarrhea | | | | | | | | |  |
| **Reference standard** | Percent of post rehydration weight gain (moderate dehydration ≥5% change) | | | | | | | | |  |
| **№ of studies (№ of patients)** | | **Study design** | **Factors that may decrease certainty of evidence** | | | | | **Effect Estimate** | **Confidence** | |
|  |  |  | **Risk of bias** | **Indirectness** | **Inconsistency^a^** | **Imprecision** | **Publication bias** |  |  |  |
|  | | | | | | | | | | |
| False positive rate of WHO IMCI algorithm versus pre and post rehydration weight change (moderate dehydration ≥5% change) | | | | | | | | | | |
| Nagpal (1994) | | Cross-sectional (cohort type) | Serious^a^ | Serious^b^ | -- | Serious^b^ | Untested^c^ | 23.3% (9.9%-42.2%) | Very Low | |

**Table F: Reasons for excluding studies at the full text review stage.**

| **Title** | **Study** | **Exclusion category** |
| --- | --- | --- |
| Clinical trial of glucose-oral rehydration solution (ORS), rice dextrin-ORS, and rice flour-ORS for the management of children with acute diarrhea and mild or moderate dehydration. | Molina 1995 | Exclusion reason: Wrong patient population; children not wasted/results not stratified by wasting |
| Rice-based rehydration solution: An alternative to glucose-based solutions in acute diarrhea in malnourished children | Razafindrakoto 1993 | Exclusion reason: Wrong intervention; not a comparison low osmolarity ORS to ReSoMal |
| Oral rehydration therapy (ORT) for childhood diarrhea. |  | Exclusion reason: Wrong study design; Wrong study design; Not a clinical trial (editorial) |
| Efficacy of oral rehydration solution in correcting serum potassium deficit of children with acute diarrhoea in Bangladesh. | Ahmed 1988 | Exclusion reason: Wrong study design; Wrong study design; Not a clinical trial (cohort) |
| Comparison of three oral rehydration strategies in the treatment of acute diarrhea in a tropical country | Pignatelli 2003 | Exclusion reason: Wrong patient population; children not wasted/results not stratified by wasting |
| Oral rehydration of neonates and young infants with dehydrating diarrhea: comparison of low and standard sodium content in oral rehydration solutions. | Bhargava 1984 | Exclusion reason: Wrong patient population; children not wasted/results not stratified by wasting |
| Oral therapy of neonates and young infants with World Health Organization rehydration packets: a controlled trial of two sets of instructions. | Bhargava 1986 | Exclusion reason: Wrong patient population; children not wasted/results not stratified by wasting |
| Is a low-osmolarity ORS solution more efficacious than standard WHO ORS solution? | El-Mougi 1994 | Exclusion reason: Wrong patient population; children not wasted/results not stratified by wasting |
| Efficacy and standard glucose-based and reduced-osmolarity maltodextrin-based oral rehydration solutions: Effect of sugar malabsorption | El-Mougi 1996 | Exclusion reason: Wrong patient population; children not wasted/results not stratified by wasting |
| Hypo-osmolar oral rehydration salts solution in dehydrating persistent diarrhoea in children: Double-blind, randomized, controlled clinical trial | Dutta 2000 | Exclusion reason: Wrong patient population; children not wasted/results not stratified by wasting |
| Long-term impact of oral rehydration in diarrhoea on nutrition of children in Calcutta slums. | Deb 1983 | Exclusion reason: Wrong study design; Not a clinical trial (cohort) |
| Oral rehydration in infantile diarrhoea. Controlled trial of a low sodium glucose electrolyte solution | Chatterjee 1978 | Exclusion reason: Wrong intervention; Not a comparison low osmolarity ORS to ReSoMal |
| Oral rehydration solution containing 90 millimol sodium is safe and useful in treating diarrhoea in severely malnourished children. | Dutta 1991 | Exclusion reason: Wrong study design; Not a clinical trial (cohort) |
| New oral rehydration solutions in acute diarrhea | Velasquez-Jones 1988 | Exclusion reason: Wrong study design; Not a clinical trial (cohort) |
| Study on efficacy of WHO-ORS in malnourished children with acute dehydrating diarrhoea. | Sharma 2003 | Exclusion reason: Wrong intervention; Not a clinical trial (cohort) |
| Oral rehydration therapy in severely malnourished children with diarrheal dehydration. | Nagpal 1992 | Exclusion reason: Wrong study design; Not a clinical trial (cohort) |
| Oral rehydration solutions in the management of acute gastroenteritis among children | Dale 2004 | Exclusion reason: Wrong study design; Not a clinical trial (Review/Editorial) |
| High sugar worse than high sodium in oral rehydration solutions | Meeuwisse 1983 | Exclusion reason: Wrong study design; Not a clinical trial (Review/Editorial) |
| Controlled clinical trial on the efficacy of rice powder-based oral rehydration solution on the outcome of acute diarrhea in infants. | el-Mougi 1988 | Exclusion reason: Wrong intervention; Not a comparison low osmolarity ORS to ReSoMal |
| Efficacy of partially hydrolyzed guar gum supplemented modified oral rehydration solution in the treatment of severely malnourished children with watery diarrhea | Ashraf 2013 | Exclusion reason: Wrong intervention: Not a comparison low osmolarity ORS to ReSoMal |
| Efficacy of partially hydrolyzed guar gum (PHGG) supplemented modified oral rehydration solution in the treatment of severely malnourished children with watery diarrhoea: a randomised double-blind controlled trial. | Alam 2015 | Exclusion reason: Wrong intervention: Not a comparison low osmolarity ORS to ReSoMal |
| [Oral rehydration solutions with 60 or 90 nmol/L of sodium for infants with acute diarrhea in accord with their nutritional status]. | Faure 1990 | Exclusion reason: Wrong intervention: Not a comparison low osmolarity ORS to ReSoMal |
| Efficacy and safety of a modified oral rehydration solution (ReSoMaL) in the treatment of severely malnourished children with watery diarrhea. | Alam 2003 | Exclusion reason: Wrong intervention: Not a comparison low osmolarity ORS to ReSoMal |
| Double blind, randomised controlled clinical trial of hypo-osmolar oral rehydration salt solution in dehydrating acute diarrhoea in severely malnourished (marasmic) children. | Dutta 2001 | Exclusion reason: Wrong intervention: Not a comparison low osmolarity ORS to ReSoMal |
| Cambios electroliticos en ninos desnutridos graves deshidratados tratados con una solucion oral modificada: Universidad de Carabobo Insalud 2005 | Conde 2005 | Exclusion reason: Wrong intervention: Not a comparison low osmolarity ORS to ReSoMal |

**Table G: Comparison of oral rehydration solutions.**

|  | | | |
| --- | --- | --- | --- |
|  | **Old Standard WHO**  **ORS** | **Low osmolarity**  **ORS** | **ReSoMal** |
|  | **(mmol/l)** | | |
| Glucose | 111 | 75 | 125 |
| Sodium | 90 | 75 | 45 |
| Chloride | 80 | 65 | 70 |
| Potassium | 20 | 20 | 40 |
| Citrate | 10 | 10 | 7 |
| Magnesium | 0 | 0 | 3 |
| Zinc | 0 | 0 | 0.3 |
| Copper | 0 | 0 | 0.045 |
| Osmolarity | 311 | 245 | 300 |

**Table H: GRADE table for the benefits and harms of standard low-osmolarity ORS compared to ReSoMal for the management of dehydration without shock, cholera or profuse watery diarrhea among children (0-59 months) with wasting.** A full description of the ROB assessment is given in Appendix 4.

The certainty in all outcomes was downgrade at least one level for indirectness, because the potassium concentration in the trial’s low osmolarity ORS was higher than that of the standard WHO formulation. The hyponatremia outcome was downgraded one further level to low, for serious imprecision related to small sample size 110 children. The hypernatraemia was downgraded to a very low certainty of evidence because of very serious imprecision. Again, this imprecision is due to the small sample size, but is exacerbated by in frequency of hypernatraemia relative to more common events like hyponatremia. Time to rehydration was also downgraded to a very low certainty of evidence due to a serious risk of bias, and serious imprecision. Clinical deterioration and mortality were downgraded to a very low certainty, due to very serious imprecision. Finally, the hypokalaemia data were deemed very low certainty because they were indirect (the potassium concentration of low osmolarity ORS had been increased) and they had very serious imprecision

| **Certainty assessment** | | | | | | | | | | | | | | | | | | | | **№ of patients** | | | | | | **Effect** | | | | | | | **Certainty** | | **Importance** | |  |  |  |  |  |  |  |  |  |  |  |
| --- | --- | --- | --- | --- | --- | --- | --- | --- | --- | --- | --- | --- | --- | --- | --- | --- | --- | --- | --- | --- | --- | --- | --- | --- | --- | --- | --- | --- | --- | --- | --- | --- | --- | --- | --- | --- | --- | --- | --- | --- | --- | --- | --- | --- | --- | --- | --- |
| **№ of studies** | | **Study design** | | | **Risk of bias** | | | **Inconsistency** | | | **Indirectness** | | **Imprecision** | | | | **Other considerations** | | | **Low osmolarity ORS** | | | **ReSoMal** | | | **Relative** **(95% CI)** | | | | **Absolute** **(95% CI)** | | |  |  |  |  |  |  |  |  |  |  |  |  |  |  |  |
| **BENEFITS** | | | | | | | | | | | | | | | | | | | | | | | | | | | | | | | | | | | | |  |  |  |  |  |  |  |  |  |  |  |
| **MORTALITY** | | | | | | | | | | | | | | | | | | | | | | | | | | | | | | | | | | | | |  |  |  |  |  |  |  |  |  |  |  |
| 1 | | randomized trials | | | not  serious | | | N/A | | | serious^A^ | | very serious^B^ | | | |  | | | 0/52 (0.0%) ^C^ | | | 0/52 (0.0%)^C^ | | | RR 1.00 (0.02 to 49.47) | | | | 0 per 1000 (from 50 fewer to 50 more) | | | VERY LOW | | CRITICAL | |  |  |  |  |  |  |  |  |  |  |  |
| **DURATION OF DIARRHEA – NOT MEASURED** | | | | | | | | | | | | | | | | | | | | | | | | | | | | | | | | | | | | |  |  |  |  |  |  |  |  |  |  |  |
| 0 | |  | | |  | | |  | | |  | |  | | | |  | | |  | | |  | | |  | | | |  | | |  | | CRITICAL | |  |  |  |  |  |  |  |  |  |  |  |
| **TIME TO FULL REHYDRATION** | | | | | | | | | | | | | | | | | | | | | | | | | | | | | | | | | | | | |  |  |  |  |  |  |  |  |  |  |  |
| 1 | | randomised trials | | | serious^D^ | | | N/A | | | serious^A^ | | serious^E^ | | | |  | | | 52 ^C^ | | | 52 ^C^ | | | Standard WHO low-osmolarity ORS had significantly longer rehydration time (19.6 hours, n=52) compared to ReSoMal (16.1 hours, n=52) (p=0.036). Authors did not present measures of variation (e.g. standard deviations, 95% CI, SEs, etc.) | | | | | | | VERY LOW | | IMPORTANT | |  |  |  |  |  |  |  |  |  |  |  |
| Low osmolarity ORS studies | | | | | | | | | | | | | | | | | | | | | | | | | | | | | | | | | | | | |  |  |  |  |  |  |  |  |  |  |  |
| 2 | | observational studies^a^ | | | not serious | | | very serious^c^ | | | very serious^c^ | | serious^e^ | | | | - | | | 112 | | | - | | | 5.33 hrs +/- 1.75  4.33 hrs +/- 0.57  36 hrs | | | | | | | VERY LOW | | IMPORTANT | |  |  |  |  |  |  |  |  |  |  |  |
| **MORBIDITY OR RECOVERY FROM CO-MORBIDITY – NOT MEASURED** | | | | | | | | | | | | | | | | | | | | | | | | | | | | | | | | | | | | |  |  |  |  |  |  |  |  |  |  |  |
| 0 | |  | | |  | | |  | | |  | |  | | | |  | | |  | | |  | | |  | | | |  | | |  | | IMPORTANT | |  |  |  |  |  |  |  |  |  |  |  |
| **DURATION OF HOSPITAL STAY– NOT MEASURED** | | | | | | | | | | | | | | | | | | | | | | | | | | | | | | | | | | | | |  |  |  |  |  |  |  |  |  |  |  |
| 0 | |  | | |  | | |  | | |  | |  | | | |  | | |  | | |  | | |  | | | |  | | |  | | IMPORTANT | |  |  |  |  |  |  |  |  |  |  |  |
| **WEIGHT CHANGE – NOT MEASURED** | | | | | | | | | | | | | | | | | | | | | | | | | | | | | | | | | | | | |  |  |  |  |  |  |  |  |  |  |  |
| 0 | |  | | |  | | |  | | |  | |  | | | |  | | |  | | |  | | |  | | | |  | | |  | | IMPORTANT | |  |  |  |  |  |  |  |  |  |  |  |
| **HARMS** | | | | | | | | | | | | | | | | | | | | | | | | | | | | | | | | | | | | |  |  |  |  |  |  |  |  |  |  |  |
| **CLINICAL DETERIORATION DEFINED BY DEVELOPMENT OF ANY DANGER SIGN^1^** | | | | | | | | | | | | | | | | | | | | | | | | | | | | | | | | | | | | |  |  |  |  |  |  |  |  |  |  |  |
| 1 | | randomised trials | | | not serious | | | N/A | | | serious^A^ | | very serious^B^ | | | |  | | | 3/55 (5.8%) | | | 3/55 (5.8%) | | | RR 1 .00 (0.21-4.74) | | | | 0 per 1000 (43 fewer to 204 more) | | | VERY LOW | | CRITICAL | |  |  |  |  |  |  |  |  |  |  |  |
| **HYPONATREMIA**^F^ | | | | | | | | | | | | | | | | | | | | | | | | | | | | | | | | | | | | |  |  |  |  |  |  |  |  |  |  |  |
| 1 | | randomised trial | | | not serious | | | N/A | | | serious^A^ | | serious^E^ | | | |  | | | 1/52 (1.9%) ^C^ | | | 8/52 (15.4%) ^C^ | | | RR 0.13 (0.02-0.96) | | | | 134 fewer per 1000 (151 fewer to 6 more) | | | LOW | | --^f^ | |  |  |  |  |  |  |  |  |  |  |  |
| ReSoMal studies | | | | | | | | | | | | | | | | | | | | | | | | | | | | | | | | | | |  | |  |  |  |  |  |  |  |  |  |  |  |
| 1 | | observational study^a^ | | | not serious | | | N/A | | | very serious^c^ | | very serious^d^ | | | |  | | | 47 | | | - | | | - | | | | 8.5% | | | VERY LOW | | IMPORTANT | |  |  |  |  |  |  |  |  |  |  |  |
| Mean sodium concentration ReSoMal | | | | | | | | | | | | | | | | | | | | | | | | | | | | | | | | | | | | |  |  |  |  |  |  |  |  |  |  |  |
| 1 | | observational study^a^ | | | not serious | | | N/A | | | very serious^c^ | | very serious^f^ | | | |  | | | 15 | | | - | | | - | | | | 131.8 mmol/l  at 8hrs | | | VERY LOW | | IMPORTANT | |  |  |  |  |  |  |  |  |  |  |  |
| Mean sodium concentration low osmolarity ORS | | | | | | | | | | | | | | | | | | | | | | | | | | | | | | | | | | | | |  |  |  |  |  |  |  |  |  |  |  |
| 2 | | observational studies^a^ | | | not serious | | | serious | | | very serious^c^ | | serious^e^ | | | |  | | | - | | | 114 | | | - | | | | 134.4 mmol/l +/- 3.1  130.6 mmol/l +/- 5.1  140.9 mmol/l +/- 5.5 | | | LOW | | IMPORTANT | |  |  |  |  |  |  |  |  |  |  |  |
| **HYPERNATRAEMIA**^F^ | | | | | | | | | | | | | | | | | | | | | | | | | | | | | | | | | | | | |  |  |  |  |  |  |  |  |  |  | 140.9 +/- 5.5 |
| 1 | | randomised trial | | | not serious | | | N/A | | | serious^A^ | | very serious^B^ | | | |  | | | 1/52 (1.9%) ^C^ | | | 0/52 (0.0%) ^C^ | | | RR 3.00 (0.13-71.99) | | | | 4 more per 1000 (2 fewer to 137 more) | | | VERY LOW | | --^f^ | |  |  |  |  |  |  |  |  |  |  |  |
| **HYPOKALEMIA**^F^ | | | | | | | | | | | | | | | | | | | | | | | | | | | | | | | | | | | | |  |  |  |  |  |  |  |  |  |  |  |
| 1 | | randomised trial | | | not serious | | | N/A | | | serious^A^ | | very serious^B^ | | | |  | | | 5/52 (9.6%) ^C^ | | | 9/52 (17.3%) ^C^ | | | RR 0.56 (0.20-1.55) | | | | 76 fewer per 1000 (138 fewer to 95 more) | | | VERY LOW | | --^f^ | |  |  |  |  |  |  |  |  |  |  |  |
| Resomal studies | | | | | | | | | | | | | | | | | | | | | | | | | | | | | | | | | | | | |  |  |  |  |  |  |  |  |  |  |  |
| 1 | | | observational study^a^ | | | not serious | | | N/A | | | very serious^c^ | | | very serious^d^ |  | | | 38 | | | - | | | - | | | | 36.8% | | | VERY LOW | | | CRITICAL | |  |  |  |  |  |  |  |  |  |  |  |
| Low osmolarity ORS studies | | | | | | | | | | | | | | | | | | | | | | | | | | | | | | | | | | | | |  |  |  |  |  |  |  |  |  |  |  |
| 2 | | | observational studies^a^ | | | not serious | | | not serious | | | very serious^c^ | | | serious |  | | | - | | | 143 | | | - | | | | 3.5% | | | VERY LOW | | | CRITICAL | |  |  |  |  |  |  |  |  |  |  |  |
| Mean potassium (Resomal) | | | | | | | | | | | | | | | | | | | | | | | | | | | | | | | | | | | | |  |  |  |  |  |  |  |  |  |  |  |
| 1 | | | observational study^a^ | | | not serious | | | not serious | | | very serious^c^ | | | very serious^f^ |  | | | 15 | | | - | | | - | | | | 3.8 at 8hrs | | | VERY LOW | | | CRITICAL | |  |  |  |  |  |  |  |  |  |  |  |
| Mean potassium (Low osmolarity ORS) | | | | | | | | | | | | | | | | | | | | | | | | | | | | | | | | | | | | |  |  |  |  |  |  |  |  |  |  |  |
| 2 | | | observational studies^a^ | | | not serious | | | serious | | | very serious^c^ | | | serious |  | | | - | | | 114 | | | - | | | | 4.22 +/- 1.06  4.60 +/- 1.11  3.5 mmol/l +/- 0.3 | | | VERY LOW | | | CRITICAL | |  |  |  |  |  |  |  |  |  |  |  |
| **OVERHYDRATION** | | | | | | | | | | | | | | | | | | | | | | | | | | | | | | | | | | | |  |  |  |  |  |  |  |  |  |  |  |  |
| ReSoMal studies | | | | | | | | | | | | | | | | | | | | | | | | | | | | | | | | | | | |  |  |  |  |  |  |  |  |  |  |  |  |
| 1 | observational study^a^ | | | not serious | | | N/A | | | very serious^c^ | | | | very serious^d^ | | | |  | | | 65 | | | - | | | - | 4.6% | | | VERY LOW | | | CRITICAL | |  |  |  |  |  |  |  |  |  |  |  |  |

^1^Including: obstructed breathing, respiratory distress, cyanosis, shock, severe anemia, convulsion, severe dehydration, profuse watery diarrhea, vomiting, and/or impaired consciousness.

^A^serious indirectness: the potassium content of WHO Low osmolarity ORS was increased in this trial. .

^B^Very serious imprecision:^:^Small sample size and a rare event suggest these estimates maybe imprecise.

^C^Denominator is 52 because 3 clinical detriorations per arm were excluded from the analysis by the authors.

^D^Serious risk of bias:^:^The risk of bias is high because the trial was open label and this outcome are subjective. Three children per arm were also removed from treatment and their outcomes were not assessed.

^E^Serious imprecision: Small sample size, but outcome is continuous or less rare which will lead to higher precision that the rare outcomes noted by footnote B.

^F^These outcomes were added after approval of the protocol to aid Guideline Development Group discussion, therefore, no importance has been designated.

| **Certainty assessment** | | | | | | | **№ of patients** | | **Effect** | | **Certainty** | **Importance** |  |  |  |  |  |  |  |  |  |  |  |
| --- | --- | --- | --- | --- | --- | --- | --- | --- | --- | --- | --- | --- | --- | --- | --- | --- | --- | --- | --- | --- | --- | --- | --- |
| **№ of studies** | **Study design** | **Risk of bias** | **Inconsistency** | **Indirectness** | **Imprecision** | **Other considerations** | **ReSomal** | **Low osmolarity ORS** | **Relative (95% CI)** | **Absolute (95% CI)** |  |  |  |  |  |  |  |  |  |  |  |  |  |
| **TREATMENT FAILURE** | | | | | | | | | | | | |  |  |  |  |  |  |  |  |  |  |  |
| Direct comparison | | | | | | | | | | | | |  |  |  |  |  |  |  |  |  |  |  |
| 1 | randomised trials | serious^b^ | N/A | not serious | very serious^d^ |  | 55 | 55 | - | 5% vs 5% | LOW | CRITICAL |  |  |  |  |  |  |  |  |  |  |  |
| ReSoMal studies^d^ | | | | | | | | | | | | |  |  |  |  |  |  |  |  |  |  |  |
| 1 | observational study^a^ | not serious | N/A | very serious^c^ | very serious^d^ |  | 65 | - | - | 1.5% | VERY LOW | CRITICAL |  |  |  |  |  |  |  |  |  |  |  |
| Low osmolarity ORS studies | | | | | | | | | | | | |  |  |  |  |  |  |  |  |  |  |  |
| 3 | observational studies^a^ | not serious | serious | very serious^c^ | very serious^d^ |  | - | 177 | - | 2.3% | VERY LOW | CRITICAL |  |  |  |  |  |  |  |  |  |  |  |
| **TIME TO REHYDRATION** | | | | | | | | | | | | |  |  |  |  |  |  |  |  |  |  |  |
| Direct comparison | | | | | | | | | | | | |  |  |  |  |  |  |  |  |  |  |  |
| 1 | randomised trials | serious^b^ | N/A | not serious | serious^e^ |  | 52 | 52 | - | 16.1hrs vs 19.6hrs | LOW | IMPORTANT |  |  |  |  |  |  |  |  |  |  |  |
| Low osmolarity ORS studies | | | | | | | | | | | | |  |  |  |  |  |  |  |  |  |  |  |
| 2 | observational studies^a^ | not serious | very serious^c^ | very serious^c^ | serious^e^ |  | - | 112 | - | 5.33 hrs +/- 1.75  4.33 hrs +/- 0.57  36 hrs | VERY LOW | IMPORTANT |  |  |  |  |  |  |  |  |  |  |  |
| **OVERHYDRATION** | | | | | | | | | | | | |  |  |  |  |  |  |  |  |  |  |  |
| ReSoMal studies | | | | | | | | | | | | |  |  |  |  |  |  |  |  |  |  |  |
| 1 | observational study^a^ | not serious | N/A | very serious^c^ | very serious^d^ |  | 65 | - | - | 4.6% | VERY LOW | CRITICAL |  |  |  |  |  |  |  |  |  |  |  |
| **HYPONATREMIA** | | | | | | | | | | | | |  |  |  |  |  |  |  |  |  |  |  |
| Direct comparison | | | | | | | | | | | | |  |  |  |  |  |  |  |  |  |  |  |
| 1 | randomised trial | serious^b^ | N/A | not serious | very serious^d^ |  | 52 | 52 | - | 15.4% vs 1.9% | LOW | IMPORTANT |  |  |  |  |  |  |  |  |  |  |  |
| ReSoMal studies | | | | | | | | | | | | |  |  |  |  |  |  |  |  |  |  |  |
| 1 | observational study^a^ | not serious | N/A | very serious^c^ | very serious^d^ |  | 47 | - | - | 8.5% | VERY LOW | IMPORTANT |  |  |  |  |  |  |  |  |  |  |  |
| Mean sodium concentration ReSoMal | | | | | | | | | | | | |  |  |  |  |  |  |  |  |  |  |  |
| 1 | observational study^a^ | not serious | N/A | very serious^c^ | very serious^f^ |  | 15 | - | - | 131.8 mmol/l  at 8hrs | VERY LOW | IMPORTANT |  |  |  |  |  |  |  |  |  |  |  |
| Mean sodium concentration low osmolarity ORS | | | | | | | | | | | | |  |  |  |  |  |  |  |  |  |  |  |
| 2 | observational studies^a^ | not serious | serious | very serious^c^ | serious^e^ |  | - | 114 | - | 134.4 mmol/l +/- 3.1  130.6 mmol/l +/- 5.1  140.9 mmol/l +/- 5.5 | LOW | IMPORTANT |  |  |  |  |  |  |  |  |  |  |  |
| **HYPERNATRAEMIA** | | | | | | | | | | | | |  |  |  |  |  |  |  |  |  |  | 140.9 +/- 5.5 |
| **Direct comparison** | | | | | | | | | | | | |  |  |  |  |  |  |  |  |  |  |  |
| 1 | randomised trial | not serious | N/A | not serious | very serious^d^ |  | 52 | 52 | - | 0.0% vs 1.9% | VERY LOW | IMPORTANT |  |  |  |  |  |  |  |  |  |  |  |
| **HYPOKALEMIA** | | | | | | | | | | | | |  |  |  |  |  |  |  |  |  |  |  |
| Direct comparison | | | | | | | | | | | | |  |  |  |  |  |  |  |  |  |  |  |
| 1 | randomised trial | not serious | N/A | very serious^g^ | very serious^d^ |  | 52 | 52 | - | 17.3% vs 9.6% | VERY LOW | CRITICAL |  |  |  |  |  |  |  |  |  |  |  |
| Resomal studies | | | | | | | | | | | | |  |  |  |  |  |  |  |  |  |  |  |
| 1 | observational study^a^ | not serious | N/A | very serious^c^ | very serious^d^ |  | 38 | - | - | 36.8% | VERY LOW | CRITICAL |  |  |  |  |  |  |  |  |  |  |  |
| Low osmolarity ORS studies | | | | | | | | | | | | |  |  |  |  |  |  |  |  |  |  |  |
| 2 | observational studies^a^ | not serious | not serious | very serious^c^ | serious |  | - | 143 | - | 3.5% | VERY LOW | CRITICAL |  |  |  |  |  |  |  |  |  |  |  |
| Mean potassium (Resomal) | | | | | | | | | | | | |  |  |  |  |  |  |  |  |  |  |  |
| 1 | observational study^a^ | not serious | not serious | very serious^c^ | very serious^f^ |  | 15 | - | - | 3.8 at 8hrs | VERY LOW | CRITICAL |  |  |  |  |  |  |  |  |  |  |  |
| Mean potassium (Low osmolarity ORS) | | | | | | | | | | | | |  |  |  |  |  |  |  |  |  |  |  |
| 2 | observational studies^a^ | not serious | serious | very serious^c^ | serious |  | - | 114 | - | 4.22 +/- 1.06  4.60 +/- 1.11  3.5 mmol/l +/- 0.3 | VERY LOW | CRITICAL |  |  |  |  |  |  |  |  |  |  |  |
| **TREATMENT FAILURE** | | | | | | | | | | | | |  |  |  |  |  |  |  |  |  |  |  |
| Direct comparison | | | | | | | | | | | | |  |  |  |  |  |  |  |  |  |  |  |
| 1 | randomised trials | serious^b^ | N/A | not serious | very serious^d^ |  | 55 | 55 | - | 5% vs 5% | LOW | CRITICAL |  |  |  |  |  |  |  |  |  |  |  |
| ReSoMal studies^d^ | | | | | | | | | | | | |  |  |  |  |  |  |  |  |  |  |  |
| 1 | observational study^a^ | not serious | N/A | very serious^c^ | very serious^d^ |  | 65 | - | - | 1.5% | VERY LOW | CRITICAL |  |  |  |  |  |  |  |  |  |  |  |
| Low osmolarity ORS studies | | | | | | | | | | | | |  |  |  |  |  |  |  |  |  |  |  |
| 3 | observational studies^a^ | not serious | serious | very serious^c^ | very serious^d^ |  | - | 177 | - | 2.3% | VERY LOW | CRITICAL |  |  |  |  |  |  |  |  |  |  |  |

^a^Randomized trials where only data from one arm was relevant to our question are described here as observational studies.

^b^The risk of bias is high because the trial was open label and outcomes are subjective.

^c^These studies are indirect as they do not compare the two products, but do have data relevant to one of the solutions in the correct population of children

^d^Small sample size and a rare event suggest these estimates maybe imprecise

^e^Small sample size, but outcome is continuous slightly improving imprecision

^f^Very small sample size

^g^Indirectness is serious here because the potassium content of WHO Low osmolarity RS was increased in this trial.
