# Supplementary material for: The diagnosis and management of dehydration in children with wasting or nutritional edema: A systematic review
Source: PLOS Glob Public Health. 2023 Nov 3;3(11):e0002520. doi: 10.1371/journal.pgph.0002520 (PMC10624296; doi:10.1371/journal.pgph.0002520)
Supplement: Attachment — Submitted filename: Rebuttal letter.docx [file pgph.0002520.s003.docx]

Rebuttal letter

PGPH-D-23-00250

The Diagnosis and Management of Dehydration in Children with Wasting or Nutritional Edema: A Systematic Review

Dear Editor,

Based on the recommendations we received about the completion date of the systematic review, we have rerun the search terms and updated the manuscript accordingly. Based on the new search, for the first dehydration diagnosis review, we identified 40 new results, and 39 were excluded from the title alone. One was excluded after a full-text review since it was conducted among adults only. For the second dehydration management review, we identified six new results and all of them were excluded after the abstract/title review. We hope the revised version addresses all the concerns.
