# Supplementary material for: The diagnosis and management of dehydration in children with wasting or nutritional edema: A systematic review
Source: PLOS Glob Public Health. 2023 Nov 3;3(11):e0002520. doi: 10.1371/journal.pgph.0002520 (PMC10624296; doi:10.1371/journal.pgph.0002520)
Supplement: Attachment — Submitted filename: Response to the Reviewer_June_2023.docx [file pgph.0002520.s004.docx]

Dear the Editor,

Thank you so much for your comments and suggestion, we believe they have really help to refine the manuscript. We have tried to address the comments, and the responses to all requested revisions are provided below.

Journal Requirements:

1. Please amend your detailed Financial Disclosure statement. This is published with the article. It must therefore be completed in full sentences and contain the exact wording you wish to be published.
a. Please clarify all sources of funding (financial or material support) for your study. List the grants (with grant number) or organizations (with url) that supported your study, including funding received from your institution.
b. State the initials, alongside each funding source, of each author to receive each grant.
c. State what role the funders took in the study. If the funders had no role in your study, please state: “The funders had no role in study design, data collection and analysis, decision to publish, or preparation of the manuscript.”
d. If any authors received a salary from any of your funders, please state which authors and which funders.

Answer

Thank you so much for highlighting the details that should be incorporated. We have included all the requested information in the revised manuscript.

2. Please provide separate figure files in .tif or .eps format.

Answer

We have changed the format of the figures and uploaded revised figures with a .tif format.

Reviewer #1: Respected authors,

Thank you very much for submitting your work at PLOS Global Public Health. I have thoroughly reviewed your work, and honestly I enjoyed reading your work. Overall, the concept of your work is exceptional, but it need some improvement in presentation.

Answer

Thanks so much for your review and constructive feedback, we really appreciate your support.

I am expecting you will work on all the suggestions and will improve the quality and presentation as much as you can.
Abstract: No comments.
Introduction: No comments.
Methodology: In your methodology section, I found a lot of tables. I suggest you write your methodology according to the PRISMA guidelines for systematic/scoping review. You can visit following links:
1) http://www.prisma-statement.org/documents/PRISMA_2020_checklist.pdf
2) http://www.prisma-statement.org/Extensions/ScopingReviews
I hope this will aid you to refine your methodology.
Moreover, in your methodology do not forget to define your variables of interest.

Answer

We appreciate your suggestion of following the PRISMA checklist. We have adapted the manuscript to conform with the PRISMA checklist (See checklist attached to resubmission). We recognize that the use of tables in the methods is a little atypical, we do think they present useful information that helps the reader contextualize the manuscript and understand some of the methodological decisions we made. We believe that inclusion of the PICO table is good practice (per GRADE guidelines) and that the inclusion/criteria table helps the reader avoid confusion about the two systematic reviews.

Results:
In the result section, start a paragraph for study characteristics. If possible create a diagram, which describe how many studies assessed wasting and how many treatment interventions. Study location, study design, study setting, duration, etc.

Answer

Thanks so much for requesting these clarifications. We have added more details on the characteristics of the included studies and we have ensured the study details re included in the Table 3, Table 4 and the appendix. The following changes were made:

# *“Diagnostic review*

*Of the 2,903 articles identified, three full texts and one abstract met the inclusion criteria (Figure 1). The studies were from India (Nagpal [1992] & Nijhawan [2020]), Bangladesh (Skrable [2017]), and South Africa (Beatty [1974], Table 3). Wasting definitions and inclusion of kwashiorkor patients varied across the studies. Diagnostic review: Beatty (1974) used Boston weight-for-age percentile (<50 percentile), and Nagpal (1992) used Gomez's weight-for-age grades (grades III&IV), Skrable (2017) used mid-upper arm circumference (MUAC <12.5cm – wasted, <11.5cm – severely wasted), while Nijhawan (2020) did not define wasting. Skrable (2017) excluded children with nutritional oedema 13, while the other studies did not comment on the inclusion or prevalence of nutritional oedema.”*

# *“****Oral fluid management review***

# *Among 1,718 articles (Figure 1, Appendix 7), only six studies - two from India, two from Bangladesh, one from Mexico, and one from Venezuela (Table 4) met the inclusion criteria.20,21 Definitions of wasting and the inclusion of kwashiorkor varied across the studies. Two studies defined wasting by the WHO criteria (Kumar 2015, Conde 2005), while the remaining studies used weight-for-length or weight-for-age z-scores <-3 (Alam 2015), a Gomez criteria grade II or III (Faure 1990), a weight-for-length < 70% of the median (Alam 2003), and a weight-for-length <60% of the Harvard/Boston scale median (Dutta 2001). Faure (1990) was the only trial to include children with moderate wasting. Prevalence of kwashiorkor varied across the studies with one study excluding these children, two studies not commenting on kwashiorkor, and the remaining studies reporting a prevalence between 22% and 73%. Studies included infants and children aged 1 to 48 months.”*

Moreover, after presenting study characteristics for each review, I suggest you try to present each study measure and outcome and present its component in separate tables. Such as while presenting electrolytes level of children with moderate to severe dehydration, discuss the finding according to table.
Do not copy and paste same thing, rather than write hyponatrimia, hypernatremia, hypokalemia, hyperkalemia, and so on.
In this way, it would be easier to understand, of six studies how many studies examine sodium and potassium level, if X studies assessed sodium and potassium level then how many reported electrolytes imbalance and how many showed normal electrolytes. Same thing you need to do for other variables, such as fluid overload and treatment failure.

Answer

Thanks so much for the very detailed comment. We have accepted your input and added summary sentences under the sections of each outcome. We are trying to balance presenting each included study separately against a more summative approach organized by outcome. For example, where our primary aim was to highlight the differences between those who used low osmolarity ORS and ReSoMal, we believe it is easier for the reader if present the result by outcome.

Discussion:
I feel that while writing discussion you need to elaborate further about the diagnostic signs and also about the management.

Answer

This is a good point. To explain some of our methodological choices we included the diagnostic methods and the management under the methods sections. We have now added a few sentence to the discussion too.

*“This review found the currently recommended WHO/IMCI dehydration algorithm to have comparable performance to available alternatives, such as DHAKA and CDS, among children with wasting (algorithms defined in appendix).”*

*“WHO currently recommends the use of ReSoMal for children with severe acute malnutrition instead of low osmolarity ORS which is used for all other children.”*

Write strengths and limitation in a separate heading.

Answer

Thank you for this suggestion, we have added a separate section for the strength and limitations

Add a heading of study implication.

Answer

Thank you for this suggestion. To avoid overburdening the manuscript with subheadings we have kept the implications under the “Conclusions” subheading. We would be happy to change this if the editors feel it would be more in keeping with the journal’s style.

Looking forward to see next version.

Best regards,

Thank you
