# Supplementary material for: The diagnosis and management of dehydration in children with wasting or nutritional edema: A systematic review
Source: PLOS Glob Public Health. 2023 Nov 3;3(11):e0002520. doi: 10.1371/journal.pgph.0002520 (PMC10624296; doi:10.1371/journal.pgph.0002520)
Supplement: Attachment — Submitted filename: Response to the Reviewer_Sep_2023.docx [file pgph.0002520.s005.docx]

Dear the Editor,

Thanks so much for your suggestion! We have tried to address all the comments and questions raised and provided the answers below.

Journal Requirements:

1. Please review your reference list to ensure that it is complete and correct. If you have cited papers that have been retracted, please include the rationale for doing so in the manuscript text, or remove these references and replace them with relevant current references. Any changes to the reference list should be mentioned in the rebuttal letter that accompanies your revised manuscript. If you need to cite a retracted article, indicate the article’s retracted status in the References list and also include a citation and full reference for the retraction notice.

Response

Thanks so much for your comments! We have checked all the references, and they are correct, and none of them have been retracted. As a result, we did not change the list of references.

Reviewers' comments:

**Comments to the Author**

1. If the authors have adequately addressed your comments raised in a previous round of review and you feel that this manuscript is now acceptable for publication, you may indicate that here to bypass the “Comments to the Author” section, enter your conflict of interest statement in the “Confidential to Editor” section, and submit your "Accept" recommendation.

Reviewer #2: All comments have been addressed

2. Does this manuscript meet PLOS Global Public Health’s [publication criteria](https://urldefense.com/v3/__https:/journals.plos.org/globalpublichealth/s/criteria-for-publication__;!!K-Hz7m0Vt54!kJVSyp3iDTDmn1n-8E--bwcShiIlhpATIIytuiIjFC02ldbPfSKtW7qb8N1ZcnLZbVN2ZovlhBNc$)? Is the manuscript technically sound, and do the data support the conclusions? The manuscript must describe methodologically and ethically rigorous research with conclusions that are appropriately drawn based on the data presented.

Reviewer #2: Yes

3. Has the statistical analysis been performed appropriately and rigorously?

Reviewer #2: I don't know

4. Have the authors made all data underlying the findings in their manuscript fully available (please refer to the Data Availability Statement at the start of the manuscript PDF file)?

The [PLOS Data policy](https://urldefense.com/v3/__https:/journals.plos.org/globalpublichealth/s/data-availability__;!!K-Hz7m0Vt54!kJVSyp3iDTDmn1n-8E--bwcShiIlhpATIIytuiIjFC02ldbPfSKtW7qb8N1ZcnLZbVN2Zq6hpduz$) requires authors to make all data underlying the findings described in their manuscript fully available without restriction, with rare exception. The data should be provided as part of the manuscript or its supporting information, or deposited to a public repository. For example, in addition to summary statistics, the data points behind means, medians and variance measures should be available. If there are restrictions on publicly sharing data—e.g. participant privacy or use of data from a third party—those must be specified.

Reviewer #2: Yes

5. Is the manuscript presented in an intelligible fashion and written in standard English?

PLOS Global Public Health does not copyedit accepted manuscripts, so the language in submitted articles must be clear, correct, and unambiguous. Any typographical or grammatical errors should be corrected at revision, so please note any specific errors here.

Reviewer #2: Yes

6. Review Comments to the Author

Please use the space provided to explain your answers to the questions above. You may also include additional comments for the author, including concerns about dual publication, research ethics, or publication ethics. (Please upload your review as an attachment if it exceeds 20,000 characters)

Response

We thank the reviewer for their time and dedication to reviewing our manuscript.

**Reviewer #2: The article is well written**

Please include the statistical methods in the article.

Response:

Thanks so much for your comments! The article is a systematic review, and we did not do a meta-analysis due to insufficient quantitative evidence. As a result, there was not a specific statistical method used. We mentioned this in the article at the end of the methods section.

Results
How did the authors overcome bias?

Response

We assessed the bias in two ways. For the dehydration diagnosis review, we evaluated the bias using the QUADAS-2 tool, and for the management review, we assessed it using the ROB-2 tool. We have explained our bias assessment and reported that accordingly, both in the methods and the result sections. However, since we didn’t do a meta-analysis, there was not a specific statistical method we applied to minimize biases due to some influential estimates.

Can the results be represented in forest plot

Response

It is a great question. Again, we have a similar answer here. We don’t have an aggregated estimate calculated based on individual findings. Unfortunately, the estimates across studies were less similar and hard to merge. Our paper mainly focuses on explicitly describing the available evidence on assessing and managing dehydration among wasted children. If we had an aggregated effect size calculated based on evidence from individual studies, we could have used forest plots to display the findings.

Why only RCTs were included?

Response

In the first part of the systematic review – the diagnosis of dehydration – we incorporated all designs. However, for the management review, which mainly focused on comparing the efficacy of ReSoMal with low osmolarity ORS, we included only RCTs because RCTs primarily addressed the research question.
